# English as a foreign language teachers’ professional success in the Chinese context: The effects of well-being and emotion regulation

**DOI:** 10.3389/fpsyg.2022.952503

**Published:** 2022-08-25

**Authors:** Jian Fan, Yongliang Wang

**Affiliations:** ^1^School of College English Teaching and Research, Henan University, Kaifeng, China; ^2^School of Liberal Arts, Shinawatra University, Pathum Thani, Thailand

**Keywords:** well-being, professional success, emotion regulation, structural equation modeling, EFL teachers

## Abstract

Given the significance of teachers’ professional success in the adequacy of education, exploring the determinants of this variable appears crucial. To address this need, this inquiry inspected the role of well-being and emotion regulation in Chinese EFL teachers’ professional success. For this purpose, 357 Chinese EFL teachers were selected to answer three pre-designed questionnaires. Structural equation modeling (SEM) using Smart-PLS was implemented to analyze teacher participants’ responses. The structural model revealed a strong, favorable connection between well-being, emotion regulation, and professional success. The model also demonstrated that both well-being and emotion regulation were highly influential in Chinese EFL teachers’ professional success. The potential implications for EFL teachers, teacher educators, and educational authorities are further discussed.

## Introduction

Teachers are believed to be the main pillars of education in any instructional-learning environment, including English classes. It means that the success or failure of educational systems highly depends on teachers’ professional performance (Wang, 2017; Coombe, 2019). Put it differently, teachers’ professional success is the key to a successful educational system. The concept of teacher professional success, also known as teacher effectiveness, generally refers to the degree to which teachers meet the instructional objectives set by themselves or educational administrators (Dordinejad and Porghoveh, 2014; Wossenie, 2014). More specifically, “English as a foreign language (EFL)” teachers’ professional success pertains to the extent to which an EFL teacher is successful at transmitting English language knowledge/skills to his or her pupils (Coombe, 2014). Successful teachers, according to the “American Association of School Administrators (AASA),” are those who effectively teach the course content, manage the classroom atmosphere, and fulfill students’ academic needs (Pishghadam et al., 2019).

Given that successful teachers are the cornerstone of any effective educational system and the key to obtaining desired learning outcomes (Zhao et al., 2016; Coombe, 2019), factors contributing to teachers’ professional success need to be studied. To give an appropriate response to this necessity, many scholars have investigated the role of personal factors, including identity (Derakhshan et al., 2020a), autonomy (Nosratinia and Zaker, 2017; de Groot-Reuvekamp et al., 2018), creativity (Khodabakhshzadeh et al., 2018), and self-efficacy (Klassen and Tze, 2014; Fathi et al., 2021) in teacher professional success. Likewise, many scholars have explored the function of interpersonal factors, namely immediacy (Nayernia et al., 2020), stroke (Pishghadam et al., 2021a), and credibility (Pishghadam and Karami, 2017) in teacher effectiveness. The role of emotions and emotional factors, on the other hand, has been overlooked. To cast more light on the role of positive emotional variables in teachers’ professional success, the current investigation attempts to assess the role of well-being and emotion regulation in Chinese EFL teachers’ professional success.

Well-Being, as a positive emotional characteristic, broadly refers to the presence of favorable emotions such as joy, satisfaction, and happiness (Garg and Rastogi, 2009). Accordingly, teachers’ well-being pertains to the degree to which they are fulfilled, happy, and cheerful in classroom settings (Wong and Zhang, 2014). As put forward by Granziera et al. (2020), teachers’ well-being is subjective to their workplace and working conditions. In this regard, McCallum (2020) articulated that the behaviors of colleagues, administrators, and students, along with the workplace atmosphere, can dramatically influence teachers’ subjective and psychological well-being. Likewise, Dreer (2022) stated that supportive working conditions help teachers attain higher levels of well-being. According to Mercer et al. (2016), instructors who enjoy an optimal level of well-being can thrive in their vocation. It implies that the absence of negative emotions (e.g., anxiety, job-related stress, apprehension, dissatisfaction) is the key to teachers’ professional success (Collie et al., 2015).

Emotion regulation, as another emotional characteristic, refers to one’s capacity to manage his/her emotional experiences (Thompson et al., 2008). More specifically, teachers’ emotion regulation pertains to individual teachers’ ability to handle the positive and negative emotions they typically experience in educational contexts (Gong et al., 2013). Teachers in any instructional-learning environment experience a wide variety of positive and negative feelings (Xie, 2021): Joy when learners fully understand the course content, satisfaction when learners get high scores in their exams, disappointment at learners’ disengagement, and exhaustion when colleagues and administrators are not supportive enough. As Greenier et al. (2021) maintained, those teachers who can down-regulate the negative feelings and up-regulate the positive ones are more likely to flourish in their careers. According to Jiang et al. (2016), regulating emotional experiences empowers teachers to create a joyful atmosphere in their classes, which is critical for learners’ academic growth.

The prominent function of positive emotions in the adequacy and quality of teaching has been widely emphasized in general education (Zhang and Zhang, 2013; Li et al., 2020). Likewise, with the arrival of “positive psychology (PP)” in the language education domain, the value of positive emotions and positive emotional variables in improving teaching effectiveness has been relatively recognized (MacIntyre, 2016). The school of positive psychology, as Seligman (2019) mentioned, is centered on the pillars of “positive institutions,” “positive emotional characteristics,” and “positive experiences.” Among them, positive emotional characteristics, including but not limited to emotion regulation and well-being, can result in teachers’ flourishment, professional development, and success (Dewaele et al., 2019; MacIntyre, 2021; Wang and Derakhshan, 2021; Sun et al., 2022). It is argued that positive emotional characteristics enable teachers to come up with novel and creative ideas that are highly influential in their professional success (Wang and Guan, 2020; Wang et al., 2021). In line with this, Bardach et al. (2022) stated that successful and unsuccessful instructors vary on certain emotional characteristics. That is, those who possess positive emotional characteristics like well-being and emotion regulation are more likely to become successful in the teaching profession. Notwithstanding, the impact of these two emotional characteristics (i.e., well-being and emotion regulation) on teachers’ professional success has been disregarded by educational researchers. That is, limited attention has been dedicated to examining the consequences of well-being and emotion regulation for teachers’ effectiveness (Simbula et al., 2012; Rüppel et al., 2015; Zaki, 2018). Further, with respect to the existing literature, no empirical investigation has been devoted to the influences of these variables on teachers’ success in English language classes. To take a step toward narrowing the gaps, this inquiry seeks to assess the role of well-being and emotion regulation in Chinese EFL teachers’ professional success. The following questions guided the present inquiry:

1. Are there any significant correlations between Chinese EFL teachers’ well-being, emotion regulation, and professional success?
2. Do well-being and emotion regulation significantly predict Chinese EFL teachers’ professional success?

## Literature review

### Well-Being

The notion of well-being generally refers to “an individual’s degree of happiness and satisfaction with his/her life, career, and physical and mental health” (Garg et al., 2014, p. 265). Narrowing this to the educational context, Skaalvik and Skaalvik (2018) defined teacher well-being as the level of happiness, joy, and happiness teachers experience at work. Similarly, Gregersen et al. (2020) referred to teacher well-being as an individual teacher’s appraisal of his or her contentment in classroom contexts. There appears to be a dispute among academics on the fundamental components of teacher well-being. Ryff (1989), for instance, grouped the components of teacher well-being under seven dimensions (e.g., self-acceptance, positive relations with others, personal growth, among others). Dagenais-Desmarais and Savoie (2012), on the other hand, characterized this construct as comprising five core facets of “*Interpersonal fit at work*,” “*Thriving at work*,” “*Feeling of competency at work*,” “*Perceived recognition at work*,” and “*Desire for involvement at work.*” As put forward by Dagenais-Desmarais and Savoie (2012), “*Interpersonal fit at work*” as the first facet of teacher well-being deals with an individual teacher’s appraisal of his/her interactions with learners, colleagues, and administrators. The second dimension, “*Thriving at Work*,” is concerned with teachers’ perceptions of teaching as a desirable career in which they may flourish. The third facet, “*Feeling of competency at work*” has something to do with teachers’ assessment of their instructional knowledge. The fourth facet, “*Perceived recognition at work*,” relates to the degree to which administrators, learners, and parents appreciate teachers’ educational endeavors. Finally, “*Desire for involvement at work*,” as the name speaks for itself, refers to teachers’ inclination to devote themselves to their vocation.

To date, a multitude of studies has been done on teacher well-being and its academic consequences (e.g., Ergün and Dewaele, 2021; Kong, 2021, to cite a few). Ergün and Dewaele (2021), for instance, explored the effects of teachers’ well-being on teaching enjoyment. To accomplish this, 174 English language teachers were selected to answer two pre-designed questionnaires. The results of regression analysis uncovered that teachers’ well-being can desirably influence their enjoyment at work. In another study, Kong (2021) assessed the impact of well-being on teachers’ involvement in Chinese English classes. To do this, two scales were virtually administered to 304 EFL teachers. Considering the path analysis outcomes, the researchers reported that Chinese English teachers’ involvement is subjective to their well-being.

### Emotion regulation

Emotion regulation refers to “extrinsic and intrinsic processes that an individual goes through to evaluate, modify, or control his/her emotions to accomplish specific purposes and goals in life” (Thompson, 2008, p. 27). In an educational setting, emotion regulation refers to the various physical, psychological, and cognitive processes that instructors use to up/down-regulate their emotions in order to carry out their job-related responsibilities (Gong et al., 2013). As Karabay (2019) mentioned, teachers who effectively demonstrate positive feelings and mitigate the negative ones can make a close bond with their learners. In this regard, Littleton (2021) also maintained that instructors who skillfully navigate their emotions can effectively communicate with pupils both inside and outside the classrooms. In a similar vein, Talbot and Mercer (2018) articulated that surpassing negative feelings such as anger, exhaustion, and apprehension empowers teachers to shield themselves against any threat to their professional success.

So far, a plethora of investigations has been implemented on emotion regulation and its possible academic outcomes (e.g., Braun et al., 2020; Chang, 2020; Han et al., 2020; Bing et al., 2022, among others). In their study, Han et al. (2020) evaluated the effects of instructors’ emotion regulation on their well-being. In doing so, 643 university instructors were invited to complete two reliable questionnaires. Data analysis showed a direct association between emotion regulation and well-being. Moreover, university instructors’ emotional regulation was found to be highly beneficial for their well-being. Similarly, Braun et al. (2020) probed the function of teachers’ emotion regulation in students’ well-being. For this goal, two scales were administered among 320 elementary learners and 15 teachers. The results indicated that teachers’ emotion regulation can favorably contribute to learners’ well-being.

### Teacher professional success

Teacher professional success, also called an occupational success, broadly refers to the extent to which teachers function effectively in the workplace (Hung et al., 2007; Elizabeth et al., 2008). According to Derakhshan et al. (2020b), successful teachers are knowledgeable, principled, and skillful people who present information clearly, employ novel instructional techniques and use a variety of instructional aids. However, as Bremner (2020) noted, the qualities of effective/successful teachers are not limited to their instructional performance. To him, an effective teacher is someone who provides learners with a joyful learning environment, cares about their well-being, prioritizes their academic interests, and develops intimate connections with them. As Zhao et al. (2016) noted, a successful language teacher is competent and skilled enough to remarkably influence students’ learning outcomes and lead them toward academic success. In this regard, Pishghadam et al. (2021b) also articulated that learners’ L2 success depends largely upon the effectiveness of their instructors in that only effective instructors can inspire their pupils to attend the classes and pursue the arduous process of language learning. To them, teachers’ effectiveness, or professional success prompts language learners to survive and thrive in classroom contexts.

A review of existing literature reveals that EFL teachers’ professional success is tied to student achievement (Wossenie, 2014), student attendance (Gershenson, 2016), and student WTAC (Pishghadam et al., 2021a). Accordingly, exploring the predictors of teacher professional success is crucial. Against this backdrop, several research studies have been executed in EFL contexts to uncover the role of personal and interpersonal factors in teachers’ success (e.g., Klassen and Tze, 2014; Derakhshan et al., 2020b; Bardach et al., 2022, among others). Yet, the function of emotional factors in improving teacher effectiveness has remained elusive. To respond to this lacuna, this study seeks to assess the role of two emotional factors, namely emotion regulation and well-being, in Chinese EFL teachers’ professional success.

## Methodology

### Participants

The participants included 357 EFL teachers who were teaching English at different educational institutions in nine provinces of China (i.e., Anhui, Zhejiang, Jiangsu, Guizhou, Jiangxi, Guangdong, Shandong, Hubei, and Henan). The sample, chosen through a convenience sampling technique, comprised 60 males (16.81%) and 297 females (83.19%), ranging in their age from 22 to 58 (*M* = 38.7, *SD* = 7.95). Most teachers were greatly experienced, with teaching experience varying from 5 to 30 years. All participants had graduated in different branches of English, including *“Teaching English as a Second Language (TESL)”* (47.1%), *“English Language Literature”* (19%), *“Applied Linguistics”* (15.4%), *“English Language Translation”* (8.9%), *“Linguistics”* (2%), and the rest (7.6%). As regards their academic degrees, they hold bachelor’s degrees (*N* = 93) or above (*N* = 264). Following the ethical guidelines for educational research (British Educational Research Association [BERA], 2018), the confidentiality of demographic information was guaranteed to participants.

### Instruments

#### Teacher well-being at work

The scale of “*Teacher Well-Being at Work (TWBW)*,” validated by Dagenais-Desmarais and Savoie (2012), was used to assess Chinese teachers’ well-being in EFL classes. The TWBW encompasses five major components, including “Interpersonal Fit at Work,” “Thriving at Work,” “Feeling of Competency at Work,” “Perceived Recognition at Work,” and “Desire for Involvement at Work.” The scale is comprised of 25 items, the responses to which can vary from 0 (disagree) to 5 (entirely agree). Sample items involve “*I like my job*,” “*I feel confident at work*,” and “*I am proud of the job I have.*” In this research, a reliability index of 0.95 was reported for TWBW.

#### Emotion regulation questionnaire

The “*Emotion Regulation Questionnaire (ERQ)*” (Gross and John, 2003) was employed to measure how efficiently Chinese EFL teachers employ emotion regulation strategies in classrooms. This 10-item scale includes two main facets, namely “Cognitive Reappraisal” (6 items) and “Expressive Suppression” (4 items). The ERQ is a 7-point Likert-type scale, varying from 1 (strongly disagree) to 7 (strongly agree). Sample items encompass “*I keep my emotions to myself*,” and “*I control my emotions by not expressing them.*” The ERQ enjoyed high internal consistency in this study (α = 0.89).

#### Characteristics of successful English as a foreign language teachers questionnaire

Chinese EFL teachers’ professional success was evaluated by “*Characteristics of Successful EFL Teachers Questionnaire (CSTQ)*” (Moafian and Pishghadam, 2009). The CSTQ involves 47 items, measuring teachers’ professional success based on their “teaching accountability” (7 items), “interpersonal relationships” (7 items), “attention to all” (5 items), “examination” (3 items), “commitment” (3 items), “learning boosters” (6 items), “creating a sense of competence” (4 items), “teaching boosters” (4 items), “physical and emotional acceptance” (2 items), “empathy” (2 items), “class attendance” (2 items), and “dynamism” (2 items). The teachers rated the items on a 5-point Likert scale, from 1 (strongly disagree) to 5 (strongly agree). The reliability of CSTQ in the present study was reported to be 0.97.

### Data collection procedure

Before commencing the data collection, the participants were notified of the academic purposes of this inquiry. They were also informed that their participation in this research was entirely optional. Additionally, the consent forms were administered to participants through WeChat messenger. Then, to initiate the data collection process, three reliable self-report questionnaires (i.e., TWBW, ERQ, and CSTQ) were virtually distributed among 357 EFL teachers, using email and WeChat messenger. It took the researchers around a month to gather the participants’ responses.

### Data analysis procedure

In this investigation, the gathered data was initially screened to detect and remove problematic and erroneous answers. Then, the internal consistency of the questionnaires was computed using composite reliability and Cronbach’s alpha procedure. Subsequently, the discriminant validity of the lower order constructs was also calculated through Fornell-Larcker and HTMT tests. Additionally, SEM using Smart-PLS (version 3.3.5) was performed to assess the interrelationships of the constructs (i.e., emotion regulation, well-being, and professional success) and the function of emotion regulation and well-being in Chinese EFL teachers’ professional success. SEM is one of the innovative methods of analysis in quantitative inquiries. As has been indicated by Novikova et al. (2013, p. 146), SEM has two major advantages over traditional multivariate methods: “explicit assessment of measurement error” and “estimation of latent variables via observed variables.” As to the first advantage of SEM analysis, Byrne (2013) stated that most multivariate methods unintentionally disregard measurement error, while SEM models assess these error variance parameters for both dependent and independent variables. Moreover, concerning the second advantage of this innovative method, he explained that SEM allows for the estimation of unobserved variables from observed variables.

## Results

Before commencing the main analysis, teachers’ responses to the questionnaires were carefully reviewed to uncover and eliminate the problematic data. Fortunately, no missing responses were discovered in the collected data. Then, the patterns of gathered responses were examined. As a result, eight responses (No. 9, 15, 23, 55, 73, 110, 181, and 192) with persistence/decreasing/increasing patterns were recognized and eliminated. Following that, the standard deviations of participants’ responses were measured, and four responses with values less than 0.5 were removed. Finally, 345 responses were remained for the main analysis.

In the first phase of the main analysis, the Cronbach’s alpha and composite reliability were computed to evaluate the reliability of well-being, emotion regulation, and teacher professional success. The outcomes demonstrated that all variables enjoyed a high degree of reliability, with a Cronbach’s alpha and a composite reliability value above 0.7 (see Tables 1–3).

**TABLE 1:** Convergent validity, Cronbach’s alpha, and composite reliability of well-being.

Well-being		Convergent validity reliability				
		
		Outer loading	*t*-values	AVE	Composite reliability Cronbach’s α	
					
	Indicators	>0.708	>2.57	>0.5	>0.7 > 0.7	
Interpersonal Fit at Work (RLOC)	InterFitWrk_01	0.82	33.282	0.63	0.894	0.851
	InterFitWrk_02	0.829	33.032			
	InterFitWrk_03	0.835	35.131			
	InterFitWrk_04	0.839	36.784			
	InterFitWrk_05	0.725	10.053			
Thriving at work (RLOC)	ThrivatWrk_01			0.664	0.908	0.873
	ThrivatWrk_02					
	ThrivatWrk_03	0.846	40.373			
	ThrivatWrk_04	0.849	42.444			
	ThrivatWrk_05	0.853	49.162			
Feeling of competency at work (RLOC)	FeelCompWrk_01	0.765	17.582	0.647	901	0.863
	FeelCompWrk_02	0.856	45.735			
	FeelCompWrk_03	0.834	36.504			
	FeelCompWrk_04	0.829	26.837			
	FeelCompWrk_05	0.731	16.001			
Perceived recognition at work (RLOC)	PerRecWrk_01	0.78	27.84	0.609	0.884	0.834
	PerRecWrk_02	0.86	54.796			
	PerRecWrk_03	0.861	56.286			
	PerRecWrk_04	0.786	29.9			
	PerRecWrk_05	0.78	10.472			
Desire for involvement at work (RLOC)	DesInvlvWrk_01	0.774	27.4	0.572	0.869	0.811
	DesInvlvWrk_02	0.763	12.333			
	DesInvlvWrk_03	0.797	35.555			
	DesInvlvWrk_04	0.809	29.26			
	DesInvlvWrk_05	0.73	21.047			

**TABLE 2:** Cronbach’s alpha, composite reliability, and convergent validity of emotion regulation.

Emotion regulation		Convergent validity reliability				
		
		Outer loading	*t*-values	AVE	Composite reliability Cronbach’s α	
					
	Indicators	>0.708	>2.57	>0.5	>0.7 > 0.7	
	EmoReg_01	0.799	11.884	0.512	0.911	0.895
	EmoReg_02	0.84	13.658			
	EmoReg_03	0.731	21.391			
	EmoReg_04	0.762	7.046			
	EmoReg_05	0.787	35.982			
	EmoReg_06	0.762	15.566			
	EmoReg_07	0.762	19.407			
	EmoReg_08	0.854	53.907			
	EmoReg_09	0.727	22.249			
	EmoReg_10	0.844	51.548			

**TABLE 3:** Cronbach’s alpha, composite reliability, and convergent validity of teacher professional success.

Teacher professional success		Convergent validity reliability				
		
		Outer loading	*t*-values	AVE	Composite reliability Cronbach’s α	
					
	Indicators	>0.708	>2.57	>0.5	>0.7 > 0.7	
Teaching accountability (RLOC)	TeachAcctbility_01	0.751	28.456	0.515	0.92	0.904
	TeachAcctbility_02	0.81	14.47			
	TeachAcctbility_03	0.858	17.705			
	TeachAcctbility_04	0.814	36.27			
	TeachAcctbility_05	0.853	10.111			
	TeachAcctbility_06	0.766	26.933			
	TeachAcctbility_07	0.771	35.653			
	TeachAcctbility_08	0.891	19.461			
	TeachAcctbility_09	0.749	25.847			
	TeachAcctbility_10	0.788	36.665			
	TeachAcctbility_11	0.897	18.728			
Teaching booster (RLOC)	TeachBooster_01	0.789	32.321	0.626	0.869	0.799
	TeachBooster_02	0.892	18.456			
	TeachBooster_03	0.29	43.52			
	TeachBooster_04	0.846	49.107			
Learning booster (RLOC)	LearnBooster_01	0.872	19.954	0.53	0.909	0.879
	LearnBooster_02	0.714	21.476			
	LearnBooster_03	0.767	31.404			
	LearnBooster_04	0.818	16.337			
	LearnBooster_05	0.822	8.296			
	LearnBooster_06	0.829	38.294			
	LearnBooster_07	0.801	35.417			
	LearnBooster_08	0.834	37.027			
	LearnBooster_09	0.842	47.777			
	LearnBooster_10	0.8	37.911			
Interpersonal relationships (RLOC)	InterRelat_01	0.742	22.436	0.567	0.901	0.871
	InterRelat_02	0.845	15.523			
	InterRelat_03	0.75	25.814			
	InterRelat_04	0.87	17.571			
	InterRelat_05	0.826	43.133			
	InterRelat_06	0.816	22.704			
	InterRelat_07	0.8	33.987			
Physical and emotional acceptance (RLOC)	PhyEmAcc_01	0.873	60.828	0.601	0.745	0.723
	PhyEmAcc_02	0.868	59.89			
Availability (RLOC)	Avlbility_01	0.912	83.602	0.746	0.854	0.802
	Avlbility_02	0.814	27.298			
Attention to All (RLOC)	AttntoAll_01	0.902	46.277	0.758	0.932	0.823
	AttntoAll_02	0.823	9.808			
Familiarity with foreign language and culture (RLOC)	Fmilirity_01	0.874	62.807	0.722	0.954	0.45
	Fmilirity_02	0.84	34.816			
	Fmilirity_03	0.863	47.812			
	Fmilirity_04	0.833	34.589			
	Fmilirity_05	0.867	54.208			
	Fmilirity_06	0.838	40.039			
	Fmilirity_07	0.875	53.05			
	Fmilirity_08	0.803	34.355			

In the second phase, Fornell-Larcker and HTMT tests were performed to measure the discriminant validity of the lower-order constructs. The results of the tests indicated that the square root of “average variance extracted (AVE)” for each construct was greater than the associations of the constructs (see Tables 4, 5). This confirms the discriminant validity of the lower-order constructs (Fornell and Larcker, 1981).

**TABLE 4:** The results of Fornell-Larcker test.

ID	Latent variable	1	2	3	4	5	6	7	8	9	10	11	12	13	14
1	AttntoAll	0.87													
2	Avlbility	0.586	0.864												
3	DesInvlvWrk	0.386	0.398	0.796											
4	Emotion Regulation	0.241	0.245	0.393	0.716										
5	Familiarity	0.544	0.464	0.348	0.112	0.849									
6	FeelCompWrk	0.376	0.405	0.773	0.407	0.383	0.894								
7	InterFitWrk	0.369	0.378	0.759	0.337	0.261	0.698	0.794							
8	InterRelat	0.758	0.681	0.458	0.285	0.578	0.474	0.403	0.753						
9	LearnBooster	0.748	0.751	0.481	0.248	0.697	0.487	0.43	0.833	0.728					
10	PerRecWrk	0.367	0.371	0.775	0.34	0.362	0.784	0.734	0.423	0.473	0.78				
11	PhyEmAcc	0.67	0.563	0.408	0.336	0.45	0.425	0.364	0.664	0.688	0.381	0.775			
12	TeachAcctbility	0.694	0.678	0.495	0.288	0.693	0.499	0.437	0.653	0.656	0.458	0.696	0.718		
13	TeachBooster	0.613	0.661	0.456	0.269	0.672	0.477	0.393	0.681	0.667	0.457	0.657	0.653	0.791	
14	ThrivatWrk	0.346	0.382	0.723	0.401	0.374	0.808	0.635	0.44	0.47	0.666	0.409	0.48	0.44	0.815

**TABLE 5:** The results of Heterotrait–Monotrait test.

ID	Latent variable	1	2	3	4	5	6	7	8	9	10	11	12	13
1	AttntoAll													
2	Avlbility	0.843												
3	DesInvlvWrk	0.52	0.54											
4	Emotion Regulation	0.27	0.288	0.42										
5	Familiarity	0.677	0.563	0.392	0.209									
6	FeelCompWrk	0.491	0.533	0.819	0.412	0.422								
7	InterFitWrk	0.483	0.507	0.806	0.345	0.284	0.804							
8	InterRelat	0.585	0.875	0.547	0.284	0.638	0.545	0.464						
9	LearnBooster	0.556	0.94	0.571	0.269	0.763	0.555	0.496	0.94					
10	PerRecWrk	0.48	0.495	0.943	0.358	0.405	0.821	0.856	0.495	0.55				
11	PhyEmAcc	0.231	0.455	0.739	0.592	0.736	0.717	0.619	0.262	0.153	0.693			
12	TeachAcctbility	0.875	0.85	0.583	0.292	0.734	0.564	0.498	0.755	0.55	0.53	0.147		
13	TeachBooster	0.82	0.871	0.56	0.268	0.764	0.572	0.47	0.63	0.617	0.554	0.157	0.596	
14	ThrivatWrk	0.446	0.494	0.854	0.408	0.41	0.825	0.721	0.5	0.536	0.891	0.738	0.54	0.52

**FIGURE 1:**
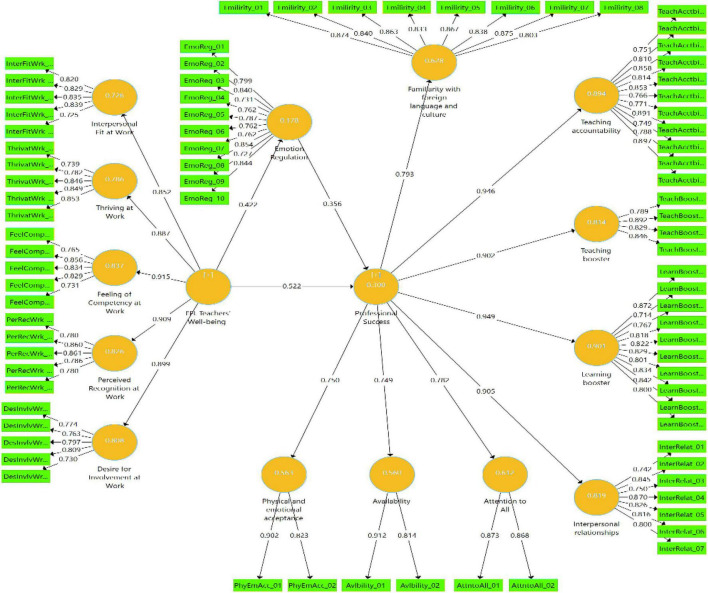
The structural model of the relationships between well-being, emotion regulation, and teacher professional success.

Additionally, to assess the associations between well-being, emotion regulation, and professional success and to analyze the role of well-being and emotion regulation in Chinese EFL teachers’ professional success, SEM was implemented through the Smart-PLS. The structural model of relationships between well-being, emotion regulation, and teacher professional success is portrayed in Figure 1.

**FIGURE 2:**
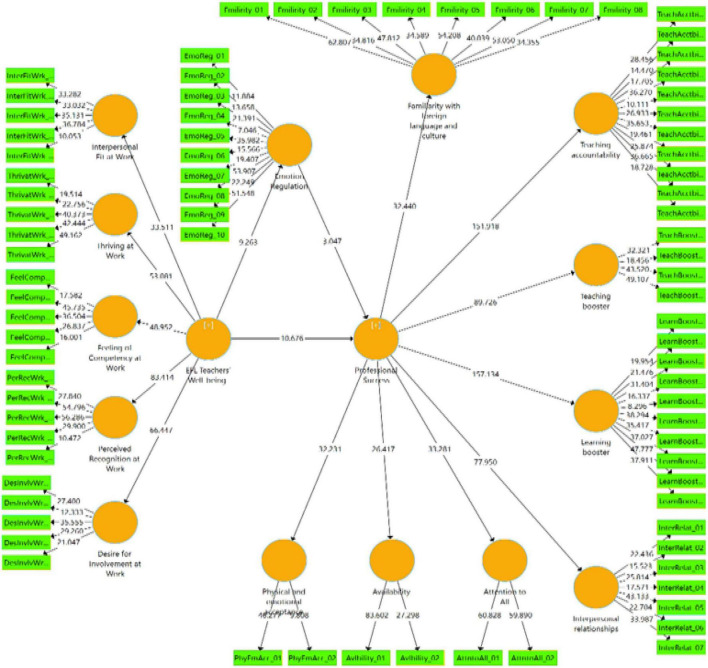
The structural model with bootstrapping results.

Finally, through Smart-PLS software, bootstrapping was performed to assess the structural model (Figure 2). The results of testing the structural model are fully presented in Table 5.

As presented in Table 6, well-being was favorably associated with teachers’ professional success (*r* = 0.300). Likewise, emotion regulation was tied to teachers’ professional success (*r* = 0.300). Moreover, a direct, positive relationship was also discovered between well-being and emotion regulation (*r* = 0.278). Finally, as Figure 1 demonstrated, both well-being (β = 0.522, *t* = 10.676, *p* < 0.001) and emotion regulation (β = 0.256, *t* = 3.047, *p* < 0.001) were found to be highly influential in Chinese EFL teachers’ professional success.

**TABLE 6:** The results of bootstrapping.

ID	H	β	*t*	*p*	f 2	*R* ^2^
H_1_	Do Chinese EFL teachers’ well-being positively predict their professional success?	0.522***	10.676	0.000	0.319	0.300
H_2_	Do Chinese EFL teachers’ well-being positively predict their emotion regulation?	0.422***	9.263	0.000	0.217	0.278
H_3_	Do Chinese EFL teachers’ emotion regulation positively predict their professional success?	0.256***	3.047	0.000	0.047	0.300
H_4_	Do emotion regulation mediates the association between Chinese EFL teachers’ well-being and professional success?	0.293***	5.190	0.000	NA	

The *** represents the level of significance.

## Discussion

The present research set out with the aim of assessing the associations between Chinese EFL teachers’ emotion regulation, well-being, and professional success. Simply said, this study aimed to inspect the degree to which the constructs are interrelated. Furthermore, this inquiry was aimed at evaluating the impacts of Chinese EFL teachers’ well-being and emotion regulation on their professional success. In fact, the present study was carried out to determine whether well-being and emotion regulation can influence teachers’ professional success in Chinese EFL classes.

As to the primary objective of this inquiry, the results of analyses revealed a direct, favorable connection, first, between well-being and professional success, and second, between emotion regulation and professional success. Additionally, a positive link was discovered between well-being and emotion regulation. The result of this study regarding the positive association between well-being and teacher professional success lends support to what Mercer et al. (2016) have argued in this regard. They articulated that teachers’ flourishment in educational settings is tied to their subjective and psychological well-being. This finding is also consistent with the ideas of Collie et al. (2015), who asserted that in the absence of negative feelings such as stress, apprehension, dissatisfaction, teachers are more likely to succeed in the teaching profession. Regarding the interrelationship between emotion regulation and teacher professional success, it is worth noting that this outcome agrees with the ideas of Greenier et al. (2021), who suggested that teachers’ occupational success is linked with their capacity to regulate the emotions up/down. Finally, the positive link between well-being and emotion regulation is on a par with some previous research undertakings in language education (e.g., Braun et al., 2020; Han et al., 2020), which discovered that one’s psychological well-being is strongly correlated with his/her emotion regulation ability.

As to the second objective of this research, the results of SEM analysis indicated that well-being and emotion regulation can considerably predict Chinese EFL teachers’ professional success. It means that EFL teachers who possess a high level of well-being and effectively handle their feelings are more likely to become successful in their career. This result accords with positive psychology premises, which proposed that positive emotional characteristics, including emotion regulation and well-being, can dramatically contribute to teachers’ professional growth, flourishment, and success (Dewaele et al., 2019; MacIntyre, 2021). Additionally, this outcome can be justified because successful and unsuccessful instructors differ on certain emotional characteristics (Bardach et al., 2022). That is, instructors who possess desirable emotional characteristics such as well-being and emotion regulation can thrive in the teaching profession. It seems encouraging to compare these outcomes with those discovered by Simbula et al. (2012) who reported that instructors’ well-being can favorably contribute to their effectiveness. These outcomes are also in congruent with Zaki’s (2018) results, which illuminated that teachers’ psychological well-being can notably raise their teaching effectiveness. Additionally, the present results appear to be in line with Rüppel et al.’s findings, which represented that psychological well-being can favorably predict teachers’ occupational success.

It goes without saying that the outcomes of this inquiry are illuminating for English teachers, teacher educators, and educational authorities. Given that emotion regulation is critical for EFL teachers’ professional success, teachers in any instructional-learning context, notably EFL classrooms, are required to regulate their feelings to become successful in their careers. Teacher educators are also expected to raise teachers’ awareness of the value of emotion regulation strategies in instructional-learning contexts. They also need to teach their teacher students how to efficiently handle their emotional experiences. Furthermore, regarding the prominence of teachers’ well-being in their occupational success, educational authorities should provide teachers with a desirable working condition in order to enhance their psychosocial and subjective well-being.

## Conclusion

This investigation was undertaken to evaluate the associations between well-being, emotion regulation, and professional success, as well as the effects of well-being and emotion regulation on Chinese EFL teachers’ professional success. As the structural model of relationships revealed, Chinese EFL teachers’ well-being, emotion regulation, and professional success are interrelated. This model also demonstrated that Chinese EFL teachers’ emotion regulation and well-being can substantially contribute to their occupational success. It is worth mentioning that these findings were limited by some important issues that can be used by other scholars to conduct related research. First and foremost, the findings were limited by the context of the study in that this investigation was fully performed in an EFL country. Since the results of this study might not be applicable to “English as a second language (ESL)” countries, future inquiries are suggested to conduct similar research in an ESL context. Second, the current study employed close-ended scales to collect data. To come up with more comprehensive outcomes, future studies are advised to utilize other data-gathering instruments, including interviews and open-ended scales. Another issue that was not addressed in this research was the effects of contextual factors. It would be interesting to assess the mediating effects of contextual factors such as age, teaching experience, academic degree, and major on the associations of the variables.

## Data availability statement

The raw data supporting the conclusions of this article will be made available by the authors, without undue reservation.

## Ethics statement

The studies involving human participants were reviewed and approved by the Henan University Research Ethics Committee. The patients/participants provided their written informed consent to participate in this study.

## Author contributions

JF was responsible for translating and distributing the questionnaire, collecting the data, and writing up the introduction, literature review and research methodology. YW was responsible for conceptualizing the questionnaire, analyzing the data, and writing up the discussion and conclusion. Both authors made substantial and direct contribution to the current study and approved the submitted version.
